# Nutritional Considerations During Major Weight Loss Therapy: Focus on Optimal Protein and a Low-Carbohydrate Dietary Pattern

**DOI:** 10.1007/s13668-024-00548-6

**Published:** 2024-05-30

**Authors:** Jeff S. Volek, Madison L. Kackley, Alex Buga

**Affiliations:** Department of Human Sciences, The Ohio State University, 305 Annie & John Glenn Ave, Columbus, OH 43210 USA

**Keywords:** Adiposity, Body composition, Dietary patterns, Low-carbohydrate, Ketogenic, Nutrition, Obesity, Weight loss

## Abstract

**Purpose of Review:**

Considering the high prevalence of obesity and related metabolic impairments in the population, the unique role nutrition has in weight loss, reversing metabolic disorders, and maintaining health cannot be overstated. Normal weight and well-being are compatible with varying dietary patterns, but for the last half century there has been a strong emphasis on low-fat, low-saturated fat, high-carbohydrate based approaches. Whereas low-fat dietary patterns can be effective for a subset of individuals, we now have a population where the vast majority of adults have excess adiposity and some degree of metabolic impairment. We are also entering a new era with greater access to bariatric surgery and approval of anti-obesity medications (glucagon-like peptide-1 analogues) that produce substantial weight loss for many people, but there are concerns about disproportionate loss of lean mass and nutritional deficiencies.

**Recent Findings:**

No matter the approach used to achieve major weight loss, careful attention to nutritional considerations is necessary. Here, we examine the recent findings regarding the importance of adequate protein to maintain lean mass, the rationale and evidence supporting low-carbohydrate and ketogenic dietary patterns, and the potential benefits of including exercise training in the context of major weight loss.

**Summary:**

While losing and sustaining weight loss has proven challenging, we are optimistic that application of emerging nutrition science, particularly personalized well-formulated low-carbohydrate dietary patterns that contain adequate protein (1.2 to 2.0 g per kilogram reference weight) and achieve the beneficial metabolic state of *euketonemia* (circulating ketones 0.5 to 5 mM), is a promising path for many individuals with excess adiposity.

**Graphical Abstract:**

Created with Biorender.com.

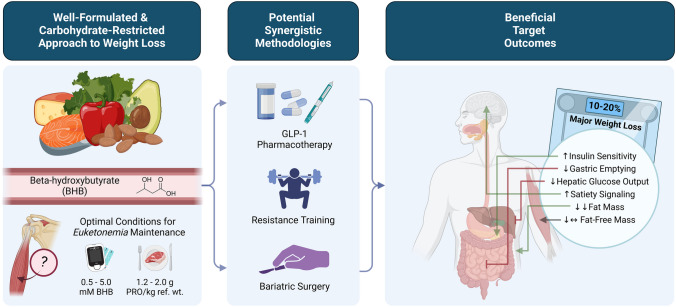

## Introduction

### Purpose

The primary objective of this review is to highlight the fundamental importance of nutrition during caloric restriction for promoting healthy weight loss. When calories are significantly restricted to induce major weight loss – which often occurs after bariatric surgery or with use of very low-calorie diets and glucagon-like peptide-1 (GLP1) analogues – maintaining muscle mass and consuming nutrient dense foods to ensure adequate essential micronutrient intakes is a high priority. In this review we examine the scientific evidence addressing nutritional considerations in maintaining lean tissue, functionality, and health during significant weight loss. This includes a focus on the optimal quantity and quality of protein necessary to achieve nitrogen balance and maximize lean tissue and health. We also review the unique role of low-carbohydrate and ketogenic dietary patterns on lean tissue, visceral adipose tissue, and other health markers during weight loss, as well as the role of exercise, including resistance training.

### Scope of the Obesity Problem

The prevalence of obesity in the United States has more than tripled over the last half century and indicate that approximately 4 out of 10 adults are classified as obese (i.e., BMI ≥ 30 kg/m^2^) and an additional one-third are overweight (i.e., BMI 25 to < 30 kg/m^2^) [1]. Thus, about 3 out of 4 adults or more than 150 million people in the U.S. have a weight problem. Childhood obesity rates are increasing at an even greater rate. Given the close association between obesity and diabetes, it may not be surprising that half of U.S. adults (> 100 million people) are either prediabetic or diabetic [2] and only a very small percentage of Americans are in good metabolic health [3]. Besides the stigma, personal suffering, and reduced health span associated with obesity and diabetes, these conditions also inflict an extraordinary financial burden on our economy with over $400 billion spent annually on managing diabetes alone, which reflects one-fifth of all healthcare expenditures and is more than all cancers combined [4]. The obesity and diabetes epidemics are not isolated to the Unites States; it is a world-wide problem. Sadly, there are few signs of stemming the obesity crisis. By 2030 nearly 1 in 2 adults in the U.S. are projected to be obese [5]. On a more promising note, the results of clinical trials indicate that bariatric surgery [6], very low-carbohydrate ketogenic diets [7, 8], and the recently developed GLP1 receptor agonists [9] have the most potent ability to produce substantial weight loss, but all requite proper attention to diet formulation to be successful.

### Nutrition is Uniquely Different than Other Weight Loss Options

For many reasons, diet is a cornerstone for weight loss therapy. Notably, diet is the only intervention that is essential (i.e., it is not optional). People with excess adiposity can choose to not have invasive surgery, engage in exercise, or take medications. But they must consume a diet and make decisions every day on what type of foods to consume. Moreover, just about every other weight loss approach, including surgery and medications, are viewed as an adjunct to nutritional counseling focused on caloric restriction.

Another unique aspect of nutrition is that it can interfere with or augment outcomes of other weight loss strategies. For example, a poorly formulated diet (e.g., deficient in protein, minerals, or some other essential nutrient) may attenuate or cancel out any positive effects of exercise or anti-obesity medications on weight loss. On the other hand, a well-formulated nutrition plan may synergize with an exercise regimen, lessen side effects of surgery, or minimize dosage of anti-obesity medications to facilitate long-term weight loss and health benefits.

The substantial weight loss achieved from bariatric surgery or GLP-1 receptor agonists is often associated with significantly decreased energy intake, as is also the case with very low-calorie or semi-starvation diets (< 800 kcal/day) in the medical management of obesity [10, 11]. The greater the degree of caloric restriction and weight loss, the more likely nutritional deficiencies occur. The new gastrointestinal physiology that accompanies bariatric surgery, and the common gastrointestinal reactions associated with GLP1 receptor agonists, also increase the likelihood of inadequate essential nutrient intake [12].

There is also the issue of weight regain, which happens frequently with all weight loss approaches, especially if individuals discontinue GLP1 receptor agonist therapy [13••, 14]. For all these reasons, nutrition must be viewed as the foundation of safe, effective, and sustainable weight loss and maintenance. Traditional weight loss strategies span from lifestyle behavioral and metabolic interventions (exercise training and diet interventions) to medical approaches that include surgery and pharmacotherapy, and various combination of these approaches. The evidence supporting these weight loss approaches is briefly reviewed next.

For this narrative review, we conducted a non-systematic literature search on PubMed and Google Scholar of articles spanning from their inception to March 2024. The Medical Search Header (MeSH) keywords used were “Glucagon-Like Peptide-1 Receptor Agonists”, “Weight Loss”, “Body Composition”, “Nitrogen Balance”, “Dietary Proteins”, “Diet, Carbohydrate-Restricted”, “Diet, Ketogenic”, “Diet, Fat-Restricted”, “3-Hydroxybutyric Acid.” These terms were combined using the Boolean operators OR and AND. Other sources were identified by manually examining and extracting the reference lists of related articles and reviews. All authors independently screened titles, abstracts, and full-text articles, with discrepancies resolved through internal collaboration.

## Relative Sucess of Different Weight Loss Approaches

### Bariatric Surgery

Bariatric surgery, which includes various invasive surgical procedures, has proven to be uniquely effective at promoting significant short-term and sustained (20-yr) weight loss [6]. According to one recent review, average weight loss at a single center at 1, 10, and 20-yr was 18, 20, and 22%, respectively [15]. There is also evidence that bariatric surgery improves obesity-related comorbidities including diabetes [16] and cardiovascular disease [17]. Bariatric surgery is generally safe, but reporting of side effects lacks rigorous quality. Early problems include leaks, stenosis, bleeding, GERD, and thromboembolic events, whereas late-complications include band erosion, obstruction, gallstone disease, Dumping syndrome, ischemia, anemia, calcium loss, kidney stones, and osteoporosis [6]. Bariatric surgery also has a substantial need for re-operative surgery [15], is expensive, and requires individuals have a BMI > 40 kg/m^2^ without accompanying disease or ≥ 35 kg/m^2^ with an accompanying disease. Thus, it is not an option for those with less severe forms of obesity.

### Pharmacotherapy

The history of drugs for obesity has mostly been marked by failure. There are only a few currently FDA-approved anti-obesity medications and – except for GLP1-analogues – they produce only moderate weight loss (< 10%) and have adverse side effect profiles [18]. In contradistinction, the recently FDA-approved GLP1 receptor agonists Semaglutide (Wegovy) and Tirzepatide (Zepbound) are associated with average weight loss similar to that of bariatric surgery (i.e., ~15 to 25%) [9] with fewer side effects and possibly many other benefits beyond obesity [19]. This degree of effectiveness represents a major advancement in the treatment of obesity, but it should be noted these drugs do not address the fundamental causes of obesity and they do require consumption of a properly formulated diet. If GLP1 receptor agonists are discontinued then weight regain occurs [13••, 14], emphasizing the need for properly formulated nutrition plans, such as carbohydrate restricted diets, that can maintain weight loss after GLP1 use [8].

There is increasing anecdotal evidence that much of the weight loss with GLP1 receptor agonists may be derived from muscle mass, although direct evidence from most large trials do not include outcome measures of body composition to substantiate this concern. In a subset of obese participants (*n* = 95) in the STEP 1 semaglutide trial who had dual-energy X-ray absorptiometry (DXA) scans performed pre- and 68-wk post intervention, total soft tissue loss was 13.6 kg and lean mass loss was 5.3 kg, or 39% of the weight loss [13••]. This represents a significant loss of muscle, some of which would be expected considering the magnitude of weight loss, but clearly there were people who lost disproportionate lean mass. Pharmaceutical companies are looking at druggable targets to address this concern, but a more relevant question is why is muscle loss happening and what non-drug measures can be taken to optimize preservation of lean tissue during major weight loss? In this regard nutrition and exercise can be force multipliers when employed appropriately.

### Diet and Exercise

Diet and exercise are considered first line approaches for weight management, but surprisingly the evidence supporting the role of exercise alone in weight loss is weak compared to other approaches [20], which often results in individual discouragement [21]. While frequent exercise training is associated with a wide range of health benefits, exercise interventions alone have a small role in weight loss [22–24]. This may seem paradoxical based on the clear increase in energy expenditure elicited by physical activity, but there are compensatory responses to exercise training that limit weight loss [25] such as increased caloric intake [26, 27] and/or decreased energy expenditure [28, 29]. There is also wide variability in how individuals translate exercise-induced energy deficits into weight loss [30], which is highly dependent on genetics [31]. Compared to endurance exercise, resistance training may offer unique benefits by increasing the proportion of fat loss while retaining lean tissue without altering total weight loss [32].

The role of diet in weight loss has a slightly better track record than exercise training, but there exists a high degree of nonconsensus related to the ideal macronutrient composition to promote healthy weight loss [33]. Most large-scale nutrition interventions for obesity have involved low-fat diets in line with the major tenets of the Dietary Guidelines for Americans and major professional medical organizations for the last four decades. The largest and longest low-fat diet study – the Women’s Health Initiative – showed that postmenopausal women randomized to a low-fat diet intervention had minor weight loss at 1-yr that was not sustained after 7-yr [34] with similar lack of benefit on breast cancer [35], type 2 diabetes [36], and cardiovascular disease [34]. In fact, there were plausibly harmful effects of the low-fat dietary pattern on future risk of coronary heart disease in the subset of women with insulin resistance [37, 38•]. Systematic analyses of long-term weight loss trials with low-fat diets have not shown a benefit over other dietary approaches [39].

Many studies over the last two decades have compared low-fat to low-carbohydrate dietary patterns on weight loss. Summarizing this large body of literature, low-carbohydrate diets are at least as effective as low-fat diets for weight loss, and often outperform them in the short-term [40, 41]. Low-carbohydrate diets appear to be especially superior to low-fat diets in individuals who are insulin resistant [42, 43]. Long-term weight loss beyond 1-yr generally shows less disparity between diets of any particular macronutrient distribution, but this is likely driven by the relative high rate of noncompliance and attrition, which could be due to several factors agnostic to diet composition.

### Holistic Approaches

It is common to combine dietary and exercise interventions, which tend to produce greater weight loss than either approach alone [20] and help preserve muscle mass during weight loss [32]. However, long-term weight loss success is still moderate even when intensive behavioral strategies are employed. For example, the Look AHEAD trial randomized over 5,000 overweight individuals with type 2 diabetes to an intensive lifestyle intervention (ILI: low-fat diet, exercise, behavior modification, meal replacements, etc.) or standard diabetes support. Although there was nearly a 9% weight loss at 1-yr in the ILI group, weight regain occurred over the next several years with an average weight loss of ~5% in ILI compared to ~2% in the control group after 8-yr when the study was halted early due to lack of efficacy [44].

The role of diet pattern prior to and after surgery has also been examined with some evidence pointing toward superior effects of a pre-bariatric surgery low-calorie ketogenic diet over a Mediterranean and other diet approaches [45, 46]. A ketogenic diet may also have benefits post-bariatric surgery, especially in those who have a poor response or weight regain [47, 48].

It is challenging to compare the relative magnitude of expected weight loss among various approaches given the massive number of studies, but we have attempted to provide a representative sampling of expected percent weight loss associated with different strategies in Table 1.

**Table 1:** Comparison of average weight loss using various approaches

**Weight Loss Approach**	**Mean Weight Loss (% Change from Baseline)**	
	**0.5 to 2 Years**	**3 to 10 Years**
**Bariatric Surgery**		
All Bariatric Procedures	18–20% [15]	19–20% [15]
**GLP-1 Analogues**		
Liraglutide (Saxenda)	3–8% [9]	6% [49]
Semaglutide (Wegovy)	10–17% [9]	
Tirzepatide (Zepbound)	15–25% [9]	
**Exercise Alone**		
	2% [50]	
	1–2% [51]	
	1% [52]	
	1–3% [53]	
	4–6% [54]	
	1% [55]	
	2% [56]	
**Diet Approaches**		
Low-Fat (LF)	3% [34]	< 1% [34]
	9% [55]	
	10% [56]	
Very Low-Carbohydrate (LC)	10–12% [7]	8% [57]
LF versus LC	LF 2%; LC 6% [58]	
	LF 4%; LC 9% [59]	
	LF 12%; LC 15% [60]	
	LF 3%; LC 2% [61]	
	LF 4–5%; LC 7–10% [62]	
	LF 3%; LC 5% [63]	
	LF 1%; LC 4% [64]	
	LF 2%; LC 4% [65]	
	LF 7%; LC 12% [66]	
**Diet + Exercise**		
LF + Exercise	9% [44]	5% [44]
	6–8% [67]	2–4% [67]
	11% [55]	
	11% [56]	

## Nutritional Considerations During Major Weight Loss

### Dietary Protein

Protein is considered an “essential” nutrient because some of the constituents of protein, amino acids, must be obtained from the diet to prevent deficiency symptoms. In nature, there are nine essential amino acids, two essential fatty acids (linoleic acid and α-linoleic acid) and no essential carbohydrates. Resupplying the amino acid pool with an adequate quantity, and quality, of protein is necessary to prevent excess loss of lean mass during weight loss. Following is a brief discussion of issues relevant to determining the optimal quantify, quality, and timing of protein intake during major weight loss.

#### Fat-Free Mass

The fat-free mass (FFM) compartment comprises skeletal muscle, bones, organs, and extracellular fluid. Skeletal muscle constitutes ~40% of adult body weight and half of the physiological amino acid pool [68]. Preserving, and in some cases augmenting, FFM in the context of major weight loss is a priority because it has a dominant role in determining total daily energy expenditure [69, 70], physical strength and activities of daily living [71, 72], lifespan [73], and many other bodily functions conducive to well-being.

FFM exists in a state of dynamic equilibrium whereby a fine balance of remodeling processes between protein synthesis and breakdown – together referred to as “protein turnover” – are required to maintain organ integrity, synthesize neurotransmitters and hormones, and produce glucose (i.e., gluconeogenesis) during prolonged fasting or to a lesser extent during a ketogenic diet [74]. A healthy adult can turnover ~5.0–6.0 g protein/kg per day [75], or in other words traffic nearly half a kilogram of protein per day between diet and FFM-stored amino acids to sustain homeostasis. These remodeling processes are thus protein intensive, and hence require adequate protein intake to modulate FFM positively.

There are two main physiological mechanisms that can augment the skeletal muscle component of FFM. De novo skeletal muscle tissue can be synthesized via satellite cell differentiation – a set of specialized cells located proximally to skeletal muscle basal lamina – that can develop into new muscle fibers (i.e., hyperplasia) in the presence of robust stimuli, such as resistance training or injury. Single muscle fibers can also increase in diameter (i.e., hypertrophy) and thereby increase their total cross-sectional area when exposed to the same stimuli, all whilst preserving the original number of fibers [76]. Whereas hyperplasia is largely influenced by epigenetic factors, hypertrophy can be rapidly modulated by resistance training and sustained by adequate protein manipulations, highlighting features that are warranted from lifestyle and pharmaceutical interventions.

#### Quantification of Fat-Free Mass

A gross estimate is that for every kilogram of body weight lost, approximately three-fourths originate from fat mass (FM) and one-fourth from FFM (3:1 ratio) [77]. However, many modulating factors can affect this ratio such as genetics, diet, training status, concurrent resistance training, weight-loss magnitude, and body composition model assumptions [78]. To accurately evaluate the composition of weight loss, non-invasive imaging techniques have advantages due to their excellent sensitivity and accuracy over metabolite excretion and anthropometric measurements, including discerning discrete changes between tissue compartments [79, 80]. Despite these advantages, all body composition methods have limitations and assumptions that make accurate determination of FFM during weight loss difficult.

Dual-energy X-ray absorptiometry (DXA) is often used as the gold standard to assess fat and lean mass, and bone density responses in many diet and training studies [79, 80]. Despite having advantages over other common body composition methods (e.g., underwater weighing, skin folds, bioelectrical impedance, air displacement plethysmography, etc.), an important limitation of DXA lies in its FFM algorithm, whereby fluid is calculated as lean tissue [81]. Thus, DXA underestimates lean mass under conditions where fluid may be lost (e.g., after profuse sweating, onset of a ketogenic diet, glycogen-depleting exercise, heavy diuretic use, etc.) or vice versa overestimates lean mass when fluid is retained (e.g., edema, carbohydrate loading, etc.). This limitation may be circumvented using other advanced imaging techniques less sensitive to fluid shifts, such as magnetic resonance imaging (MRI), but nevertheless problematic for studies comparing diets, especially ketogenic diets, expected to modulate fluid storage by altering carbohydrate intake, notably in the first few weeks of diet initiation [82–85].

#### Protein Requirements

Dietary protein is the only nitrogen-containing macronutrient, and thus requirements for protein are often determined using the nitrogen balance method, which calculates nitrogen ingested and subtracts nitrogen excreted [86, 87]. Assuming that ~16% of dietary protein is nitrogen by weight, daily nitrogen balance can be calculated mathematically by subtracting nitrogen measured in a 24-h urine collection plus nitrogen loss through sweat and feces as follows [88]:

$$\mathit{Nitrogen\;Balance}\;=\;\mathit{Protein\;Intake}\;\left({g}/{day}\right)* 0.16\;-\;[\mathit{Urine\;Nitrogen\;Excretion}\;(g/day)\;+\;4]$$

Positive nitrogen balance reflects greater protein synthesis than degradation and FFM preservation or gain, whereas negative nitrogen balance reflects greater protein breakdown than synthesis and net loss of FFM over time. The current recommended dietary allowance (RDA) for maintaining nitrogen balance is 0.8 g protein/kg/day, which translates to about 10%–15% of the total daily energy expenditure. This threshold value was determined by the USDA and WHO/FAO based on 24-h urinary nitrogen excretion patterns to achieve nitrogen balance in the majority of healthy sedentary adults [89, 90]. Extensive debates on the adequacy of 0.8 g/kg protein have permeated the literature due to concerns of poor ecological validity in athletes, older adults, or clinically ill patients, and inherent limitations in nitrogen excretion estimations owing to integumentary and fecal nitrogen loses [89, 91, 92].

Relevant to this review, the adequacy of the RDA for protein in the context of major weight loss is also dubious, as studies have indicated skeletal muscle protein synthesis is down-regulated and protein degradation accelerated during energy deficits [93]. A ~10% decrease in daily calories has been shown to increase protein requirements 50% [94]. Complicating interpretation of protein requirements during weight loss is the fact that the mechanisms regulating protein turnover are modulated by the degree of energy deficit and time. It is well documented that the amount of protein required to maintain nitrogen balance during energy deficit is reduced over time as adaptive mechanisms act to preserve lean tissue [83, 95, 96]. Another complicating factor is the understanding of “anabolic resistance,” an inflammatory-mediated mechanism that disrupts normal protein synthesis relative to normal-weight controls [97, 98].

Protein intake guidelines build on more robust evidence from detailed amino acid oxidation methods (often involving a tracer amino acid such as ^13^C-lysine/leucine or ^2^H_5_-phenylalanine) and dose-response clinical trials [91, 92, 99–103]. Collectively, an optimal dietary protein intake for weight-loss is approximately two-fold higher than previously estimated. A range of ~1.2 to 2.0 g protein/kg/day is supported by large empirical evidence as an optimal dietary protein target that can offset untoward proteolytic turnover and promote protein synthesis, highlighting a substantial discrepancy from the prior guidelines [92, 101, 102, 104–111].

During major weight loss, the question of whether it is best to seek a protein intake that achieves a zero nitrogen balance (i.e., equal rates of protein synthesis and degradation) or a protein intake that achieves a high net positive nitrogen balance and gain in FFM is worth considering. It has also been postulated that high protein diets may have other beneficial outcomes, begging the question of minimum versus optimal protein intake. A review of very high protein diets is beyond the scope of this review but has been done so by others [112].

Importantly, protein requirements should be calculated based on a reference or adjusted weight in people with excess adiposity because protein needs are primarily a reflection of the lean tissue and not adipose tissue. As shown in Table 2, a typical normal-weight male (Person A) may have an upper limit of reasonable protein intake of 150 g/day. But a similar height individual with obesity (Person B) would be prescribed protein up to 250 g/day using their actual weight, which is likely excessive relative to requirements needed to maintain lean tissue and functionality.

**Table 2:**
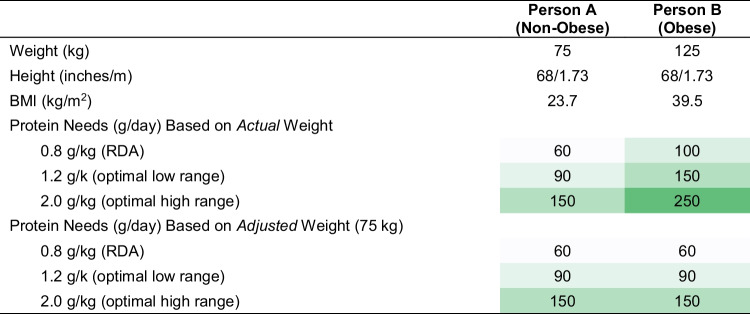
Protein adequacy vs. over-prescription

The color gradient intensity is relative to the 60 g protein dose

#### Protein Quality

The biological value of proteins is influenced by the amount of nitrogen retained in the body; a reflection of individual amino acids being incorporated into FFM. Complete dietary protein sources contain 20 amino acids, 9 of which are essential amino acids (EEA): histidine, leucine, isoleucine, lysine, methionine, phenylalanine, threonine, tryptophan, and valine. Protein quality is directly related to the presence of EAA, especially leucine, the most robust activator of mTORC1 – a master regulator of protein synthesis [113–115].

Protein sources that are limiting in EAAs are considered lower quality or incomplete proteins that can hinder the FFM growth and development in response to diet and exercise [116, 117], an effect that becomes even more important during weight loss. When consumed as intact foods, EAAs are highly abundant in all animal-derived meats and byproducts (i.e., eggs, dairy), except collagen and gelatin, and several plant sources [113, 114]. As a side note, the protein digestibility-corrected amino acid scale – a standardized range of amino acid absorption per unit of protein consumed; PDCAAS – uses eggs as the reference food for *high-biological value* determinations.

It is well-established that omnivores achieve their total protein and EAA with greater ease than vegetarians/vegans. Plant-based approaches may often provide inadequate amounts of leucine, lysine, methionine, and threonine because the amount of EAAs in plant foods is generally less compared to animal foods and can plausibly compromise protein synthesis [118, 119]. Moreover, intact plant protein sources may lack the necessary digestibility relative to equal amounts of animal proteins because plant proteins often tend to integrate within the plant fiber matrix, creating an inaccessible mesh during digestion that provides less bioavailable protein.

With the advent of protein powder formulations and the ability to decouple plant proteins from their fiber matrix, protein supplements have been able to increase EAA availability to a point where animal and plant-based powders exhibit similar kinetics with respect to their protein synthesis potential [119, 120], albeit in several instances still favoring whey [118]. Whey protein is ~50% by weight EAAs and 11% leucine [102, 119]. A quality source of whey protein will provide 25 g of protein per serving, with ~3 g from leucine. This guideline is often cited in the literature as a proxy against protein “spiking” (i.e., diluting the content with cheap, non-essential amino acids); therefore, supplementing a diet with whey during weight loss requires two major considerations: that the supplement received a reputable third-party purity certificate (i.e., NSF) and individual amino acids are listed in gram amounts on the label, which is optional.

Collectively, protein quality is guided by EAA composition, especially leucine content. Although outside the scope of this review, some qualities of dairy proteins remain to be elucidated albeit relevant to carbohydrate-restriction. Of importance, whole-eggs – specifically egg yolks – may augment protein synthesis independent of total calories and protein content, in part explained by non-caloric compounds (e.g., miRNAs) contained within the yolk fraction [121–123]. Similarly, full-fat milk (3.5%) appears to exert protein synthesis signaling properties that are beyond skim milk (0%) in the post-exercise window [124]. When viewed within a low-carbohydrate context, inclusion of full-fat dairy products is not only appropriate and encouraged but may strategically modulate protein synthesis signaling beyond fat-free items.

#### Protein Dosing and Distribution

Beyond the quantity and quality of protein, the optimal protein dose (grams per meal) and distribution (timing or frequency of intake over the day) has been a subject of research and debate over the last few decades, and in more recent discourse, the physiological upper-limit of protein digestion questioned [125]. In general, a meal that provides ~25–30 g of a high-biological value protein can adequately stimulate muscle protein synthesis due to the intrinsic connection between total protein dose and EAA content, specifically leucine, to surpass rate-limiting threshold for protein synthesis [115, 126–128]. Substituting incomplete protein sources for dairy products may lower the leucine-threshold requirement per meal (15–20 g), in part owing to the insulinogenic amino acid profile and the unique yolk/dairy matrix properties discussed earlier and in greater detail elsewhere [102, 129].

**Fig. 1:**
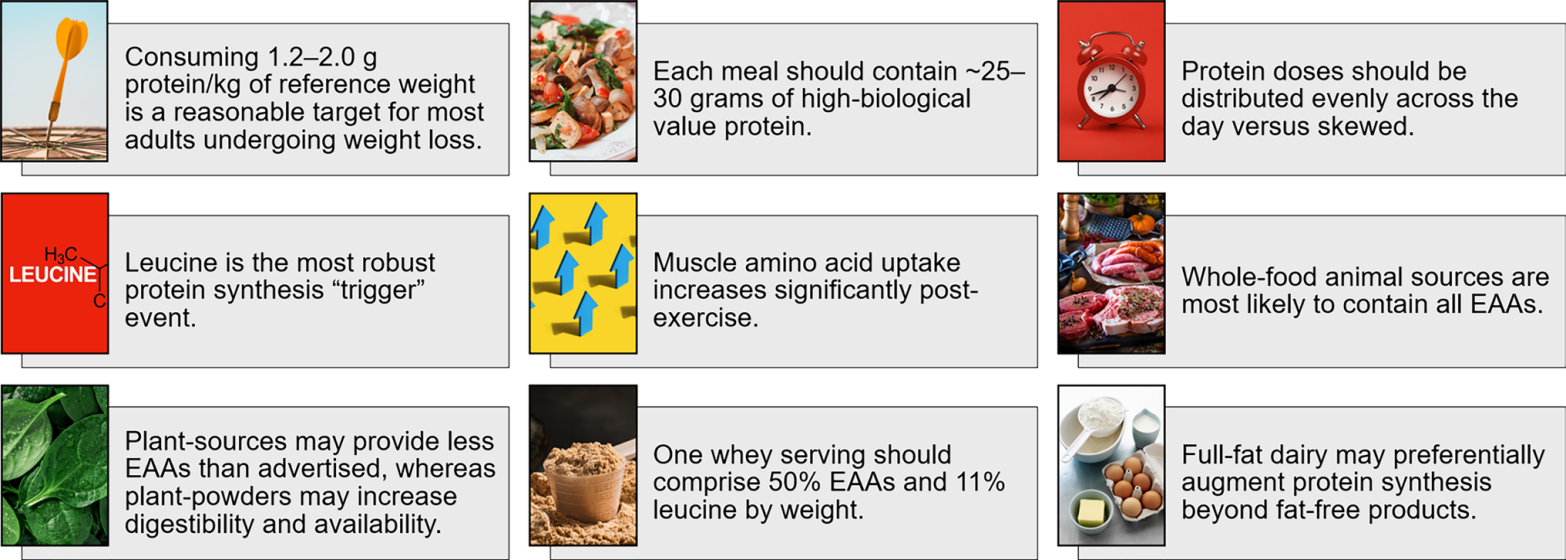
Dietary protein graphical summary

Regarding protein timing, evidence from both pre-clinical and clinical trials suggests that distributing total daily protein evenly across breakfast, lunch, and dinner can drive greater anabolic responses and FFM preservation relative to skewed intakes [111, 118, 126, 130–132]. In other words, when total daily protein is held constant, an individual who consumes at least 25–30 g of protein per meal should expect a 25% greater protein synthesis response compared to an individual who consumes a low-protein breakfast (~10 g) and a protein-rich dinner (~60 g) [132]. Collectively, it appears that overweight/obese individuals who undergo weight-loss may benefit from the protein distribution strategy presented herein [133], albeit some evidence suggests no demonstrable effects of protein distribution [134], in part explained by differences in training status (i.e., less precision required for untrained vs. trained individuals) [135]. A graphical summary of the main points discussed in this section is provided below (Fig. 1).

### Low-Fat vs Low-Carbohydrate Diets

A comprehensive discussion on various dietary patterns is beyond the scope of this review, as the permutations of nutrient combinations even within low-carbohydrate diets are vast. With the focus of this review on low-carbohydrate dietary patterns, we provide a general overview comparing and contrasting the main differences between low-fat and low-carbohydrate diets with particular attention on ketogenic diets. The reader is referred to a more detailed description of fundamental principles of a ketogenic diet [136••].

#### Definitions and Terminology

For this review, we advocate definitions of dietary patterns as proposed in a recent expert consensus paper [137], where very-low carbohydrate/high-fat ketogenic diets consist of < 10% carbohydrate or 20–50 g/day; low-carbohydrate diets consist of 10–25% carbohydrate or 50–129 g/day; and low-fat/high-carbohydrate diets consist of > 45% carbohydrate or > 225 g/day. Notably, not all low-carbohydrate dietary patterns are ketogenic, but all ketogenic diets are low in carbohydrate.

A low-fat dietary pattern is reduced in overall fat intake, especially saturated fat. Most low-fat approaches encourage the consumption of lean proteins, fruits, vegetables, and whole grains while limiting high-fat dairy products, fatty meats, and processed foods. Variations of low-fat diets may encourage primary replacement of saturated fat with carbohydrate and/or polyunsaturated fat. Most plant-based vegetarian patterns would be considered low-fat, but it is possible to also design plant-based low-carbohydrate diets. In contrast, the hallmark of a low-carbohydrate diet generally involves restricting many carbohydrate-dense foods while consuming moderate protein, and moderate- to high-fat (depending on the degree of caloric restriction). There is an emphasis on avoiding added sugars, limiting highly processed foods, and consuming nutrient-dense minimally-process whole foods without extensive packaging or a lengthy list of ingredients on the Nutrition Facts Label.

The hallmark of a ketogenic diet is limiting carbohydrate intake to < 50 g/day but can vary between people from ~20 to > 70 g/day based on their ketone levels. This level of carbohydrate restriction requires avoiding carbohydrate-dense foods such as bread, cereals, pasta, cakes/cookies, high-glycemic fruits (e.g., apples, bananas), and starchy vegetables (e.g., sweet potatoes, corn, peas, carrots). The limited carbohydrates are primarily derived from non-starchy vegetables, nuts, seeds, low-sugar/high-water content fruits (berries, avocados, olives, tomatoes), with lower amounts from full-fat dairy, whole eggs, and other protein sources. Protein needs are estimated based on 1.2 to 2.0 g/kg reference weight, usually ranging between ~80–150 g/day. Fat is prescribed to satiety which can be consumed from meats, high-fat dairy (butter, cheese, sour cream, heavy cream), avocados, oils (olive, canola, avocado, coconut, etc.), nuts and seeds. Since it is the carbohydrate and protein content that have the most impactful effect on ketone production, it is helpful to have people measure ketones to titrate the specific amounts of those nutrients that can be consumed to achieve nutritional ketosis (defined in next section). A ketogenic diet is not one-size-fits-all, as achieving nutritional ketosis is influenced by factors such as activity level, degree of insulin resistance, stress, etc.

Ensuring adequate essential vitamin and mineral intakes is a priority for all dietary patterns, especially during caloric restriction, but practically challenging for individuals to accomplish without professional support [138, 139]. Most individuals who qualify for bariatric surgery are micronutrient deficient [140]; remarkably ~99% lack adequate vitamin-D [141] and women are twice as likely than men to be iron-deficient [141]. However, poor micronutrient status can be reversed with proper supplementation [142]. Relevant to carbohydrate-restriction, the whole-food items proposed by well-formulated ketogenic diet guidelines are congruent with the micronutrient malnutrition reversal guidelines [143]. Human trials further support the idea that a ketogenic diet is micronutrient adequate in patient populations [144], and when implemented over 4-weeks in pre-operative individuals (*n* = 27) it can favorably reverse micronutrient deficiencies during major weight loss (-10%) while simultaneously improving body composition (5:1 FM:FFM kg:kg losses) [145]. Whereas the consensus is still extensively investigated [139], or obfuscated by dietary assessment methods [138, 146] and early clinical evidence [147, 148], there is recent promising data suggesting that carbohydrate-restriction is sensible to micronutrients with proper implementation and guidance.

Particularly noteworthy is the issue of sodium, which is an essential but controversial mineral. At the onset of a low-carbohydrate diet, especially a ketogenic diet, the kidneys switch from retaining sodium to rapidly excreting it [149]. In many obese individuals, this natriuresis and diuresis is associated with positive effects (e.g., rapid weight loss, less swelling, reduced blood pressure), but once this excess fluid is cleared continued loss of sodium and fluid leads to what has been termed “keto-flu”. The contracted plasma volume can manifest in dizziness, fatigue, headache, and lethargy. It also triggers the adrenal glands to release aldosterone and cortisol to retain sodium, but this occurs at the expense of losing potassium in the urine. Thus, restriction of sodium on a ketogenic diet can disrupt metabolic and hormonal homeostasis compromising sustainability and effectiveness of a ketogenic diet. Careful attention to ensuring adequate sodium as well as potassium and other minerals is necessary, which can be accomplished with normal foods, but regular consumption of broth or dietary supplements are effective as well.

Even well-intended investigators studying ketogenic therapies often fail to appreciate the challenges, principles, and nuances associated with implementing a ketogenic diet that achieves nutritional ketosis. Ketogenic nutrition is not taught to dietitians, physicians, or any healthcare professionals through traditional academic curricula, which translates into widely varying approaches used in clinical research and practice. Too often the ketogenic approach is poorly formulated and implemented either too casually or restrictive to an extreme, with poor outcomes that jeopardizes safety, acceptance, and sustainability. This is particularly true when investigators rely on chemically-formulated low carbohydrate preparations for all or most of their test diet, since these are typically deficient in multiple regards ranging from total energy to palatability rendering them unsustainable beyond a few months.

Finally, a quick note about the future of ketogenic interventions. While many ketogenic research interventions remain focused on the ketogenic diet, the recent development and commercial availability of “exogenous ketones”, which includes several different classes of compounds, now broadens the arsenal of methods to achieve physiological target ketone levels in individuals. Exogenous ketones consist of varying compositions such as direct forms of beta-hydroxybutyrate (BHB) in the form of an acid, salt, or joined with other ketone-promoting components by ester bonds. They may contain ketogenic precursors that metabolize to ketones in the liver (i.e., medium chain fatty acids), or ketone precursors that metabolize to BHB via non-classical metabolic pathways (i.e., (*R*) -1,3 butanediol).

#### Ketone Nomenclature

On an individual level, the goal of a ketogenic diet is to achieve a metabolic state of nutritional or physiological ketosis defined as circulating ketone concentrations (usually measured as beta-hydroxybutyrate, *R*-BHB) between 0.5 to 5.0 mM, which is consistently above levels demonstrated in people consuming low-fat diets.

**Fig. 2:**
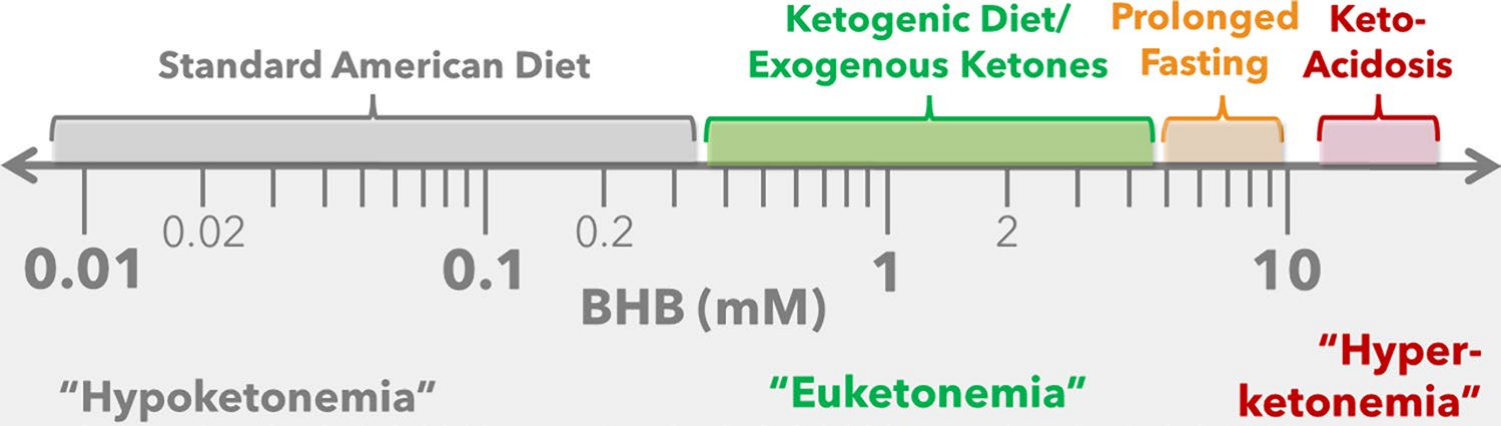
Circulating ketones, principally betahydroxybutyrate (R-BHB), span over four orders of magnitude [< 0.01 to > 10 mM]. Emerging research indicates that moving from relatively low ketone levels (i.e., "hypoketonemia") observed in people consuming a standard American diet to moderate ketone levels associated with nutritional ketosis (i.e., ~ 0.5 to 5.0 mM or "euketonemia") is associated with a myriad of health benefits

Unique among metabolites, ketones are regulated across a wide range that, when expressed in molar concentration, span a remarkable four orders of magnitude (Fig. 2). Ketones are always being produced, primarily in the liver, but the carbohydrate loads typical of low-fat or even moderate-carbohydrate dietary patterns significantly suppress ketogenesis to levels that are often < 0.1 mM and remain < 0.3 mM even after an overnight fast. The carbohydrate restriction and moderate protein consumption associated with a ketogenic diet result in markedly accelerated adipose tissue lipolysis and ketogenesis that manifest in nutritional ketosis (0.5 to 5.0 mM). Ketone levels rarely exceed 5.0 mM on a ketogenic diet due to physiological adaptations such as increased ketone utilization and inhibition of ketone production. The normal negative feedback is impaired in type 1 diabetics who do not produce insulin and are not taking adequate exogenous insulin. Thus, in uncontrolled type 1 diabetes or other rare insulin insufficient conditions, there is a possibility of developing keto-acidosis where ketone levels can exceed 20 mM.

To distinguish between these widely varying ketone levels, we propose new terminology that more accurately captures the understanding of human ketone physiology across the evolutionary spectrum, lifespan, and role in health and disease. This nomenclature aligns with the accepted terms used to describe relative glucose levels which are “hypoglycemia”, “euglycemia”, and “hyperglycemia”; corresponding to “too low”, “normal”, and “too high”, respectively. The etymology of the prefix “eu-” originates from ancient Greek and translates to “good” or “beneficial”, whereas “emia” denotes “a substance is in the blood”. Thus, we propose a new term *“euketonemia”* − an amalgamation of “eu-” and “ketone” and “emia” − to describe the desirable range of ketones, often referred to as nutritional ketosis, shown in a burgeoning number of published studies to be beneficial or therapeutic [150]. In distinction to health-promoting euketonemia, higher levels of ketones akin to keto-acidosis should be designated *“hyperketonemia”* and lower levels of ketones such as those present when consuming high-carbohydrate dietary patterns as *“hypoketonemia.”*

In support of adopting this nomenclature, mild ketosis was the normal state for humans prior to the agricultural revolution, which represents the vast majority of human history. Humans have a strong evolutionary adaptation to ketones. The brain [151] and many other tissues like the heart [152] prefer ketones, oxidize them in proportion to their circulating level, and function better when using them as fuel [153, 154]. As further evidence of euketonemia being a natural healthy state is the observation that amniotic fluid is euketonemic (average BHB 0.6 mM) [155] and many breast fed neonates are in nutritional ketosis [156]. In the case of T2D, low-fat/high-carbohydrate diets rarely reverse the metabolic disorder, whereas ketogenic diets often do [7]. Thus, one perspective viewed through the lens of T2D is that therapeutic diets that achieve euketonemia correct the metabolic state of hypoketonemia. Additional evidence supporting euketonemia as a health-promoting and therapeutic state is expanded on in later sections.

### Ketone Physiology

The three primary ketone bodies produced primarily in the liver from partial breakdown of fatty acids are BHB, acetoacetate (AcAc), and acetone. Ketone production (i.e., ketogenesis) is upregulated when hepatic glycogen and insulinemia are maintained invariably low, which occurs during a low-carbohydrate, moderate-protein diet, resulting in increased presence of principally BHB into a physiological range of nutritional ketosis or euketonemia [136••, 157, 158]. Circulating ketones are then transported to extrahepatic tissues where they serve as important energy substrates and epigenetic signaling molecules [159, 160]. During a ketogenic diet or prolonged fasting, the liver likely produces 75–150 g ketones per day [161, 162] which serves as an important alternative fuel for the brain, heart, and skeletal muscle.

Much of what is known about ketone metabolism was elucidated half a century ago through elegant experiments performed by George Cahill and his trainees who primarily studied prolonged starvation in humans [161]. Among many discoveries, was the crucial role ketones have in ensuring an adequate supply of fuel to the brain during periods of low carbohydrate availability [160]. Over the last decade, new aspects of ketone physiology have emerged. Notably, ketones are now recognized as uniquely efficient in that they generate more ATP per unit oxygen than other metabolites [163]. Ketones have been discovered to be important signaling molecules [150] working through multiple mechanisms (e.g., covalent protein modification, non-covalent enzyme regulation, receptor agonism/antagonism) that positively influence a wide range of cellular functions such as gene expression, inflammatory pathways, and immunomodulation [164, 165]. These new discoveries have led to a surge in the basic and applied study of ketogenic interventions across a wide swath of health and disease.

### Very-Low-Calorie Diets

Before we discuss comparative research on low-fat versus low-carbohydrate weight loss diets, a brief note is warranted on very-low-calorie diets (VLCDs) or semi-starvation diets, usually defined as < 800 kcal/day [166]. These diets are medically supervised in obese individuals to elicit significant weight loss (1–2 kg/week), preserve FFM, and monitor symptoms of dehydration, mineral losses, or other health complications [167, 168] often before considering bariatric surgery.

VLCDs have profound effects on nitrogen balance that in some instances precede dietary protein considerations [94, 169]. In a hypothetical scenario, the average stature woman and man in the United States with a BMI of 40 (“severe obesity”) weighs ~105 and ~120 kg, respectively [170]. A conservative protein intake of 1.2 g/kg/day, the low-end for healthy active adults, would provide them 125–150 g of dietary protein per day, or ~63–72% of their total energy intake on a VLCD. In reality, protein requirements on VLCDs are closer to 1.0 g/kg of reference weight (i.e., target weight scaled to body height) [171, 172]; therefore, protein goals scaled to ideal vs. starting weight will always be lower for obese individuals relative to overweight persons undergoing modest weight loss rates (< 1 kg/week). For brevity purposes, the consensus on protein intake for VLCDs is ~60 to 90 g/day to prevent excess nitrogen excretion [28, 171, 173–175].

There is evidence that adding carbohydrates to suppress ketone formation during VLCD protocols could hinder nitrogen balance. First, adding carbohydrates detracts the total available calories that can be reserved for protein, thereby compromising the key macronutrient conducive to FFM preservation [110, 176]. Second, evidence suggests that achieving a level of nutritional ketosis may assist nitrogen preservation during VLCDs, especially when minerals are replenished and hydration is tightly monitored [28, 85]. Ketone bodies during severe energy restriction have demonstrable effects on nitrogen balance in as little as ~2 weeks after diet initiation, and when BHB is constitutively sustained [28], compared to ~4 weeks when BHB is absent [173]. Importantly, for the majority of the population the central challenge with VLCDs lies within the behavioral and nutritional efforts wherein maintaining the target weight after weight-loss, and the lifelong countermeasures to prevent weight-regain, are the main priorities [171, 177].

### Effects on Weight Loss

There are now several published comparative studies between low-fat and low-carbohydrate dietary patterns of less than 130 grams per day of carbohydrate ranging from a few weeks to 2-years in duration. It is clear that individuals can lose weight on a variety of diets varying in macronutrient composition. The results of multiple recent meta-analyses indicate that low-carbohydrate dietary patterns do at least as well, and often better, at promoting weight loss and certain risk markers for cardiovascular disease when compared with low-fat dietary patterns [40, 41, 178–180]. A unique aspect of low-carbohydrate diets is that in many studies they were implemented *ad libitum* without prescribing an explicit caloric reduction, yet still elicited significant weight loss [181, 182]. Without going into detail on the many individuals trials now published, there is generally less of a discrepancy in weight loss among diets of any macronutrient composition the longer the intervention duration [183], emphasizing the need for ongoing support and selecting the most appropriate diet that works for each person. In regard to this idea of personalized or precision nutrition, it is noteworthy that low-carbohydrate diets appear to be especially superior to low-fat diets in individuals who are insulin resistant or have high insulin secretion [42, 43, 184–186], which is common in people with obesity. In regards to the challenge of the high cost and poor persistence of GLP1 therapy, there is now evidence that a low-carbohydrate diet may enable deprescription with maintenance of weight loss and clinical improvements [8].

### Effects on Body Composition

Considering both low-fat and low-carbohydrate dietary patterns can be used to achieve weight loss, a crucial question is whether the composition and distribution of weight loss is similar between diets. Ideally, weight loss strategies should target FM reduction, particularly in visceral adipose tissue (VAT), while preserving lean tissue. According to meta-analyses, low-carbohydrate diets compared with other weight loss interventions of the same duration demonstrate greater loss of total weight, fat mass, and waist circumference [187–189]. Because total weight loss is often higher on a low-carbohydrate diet, there may also be a greater amount of lean mass lost, but this is an expected response and associated with an overall maintenance of physical function and decrease in percent body fat. Discrepancies between individual studies may be attributed to factors such as inadequate protein intake, limitations in common body composition assessment methods that misinterpret water loss as a decrease in lean tissue, and insufficient sodium compensation for the natriuretic effect of carbohydrate-restriction.

In a few short-term hypocaloric diet studies, a greater loss of nitrogen has been demonstrated with ketogenic diets compared to non-ketogenic diets [190, 191], but these diets were severely energy-restricted, very short-term, and more importantly did not provide adequate sodium and potassium to account for the natriuretic effect of ketogenic diets, which has a key role in maintaining nitrogen [192]. In contrast, there may be some instances where a ketogenic diet could lead to greater preservation of lean tissue during major weight loss, especially if combined with resistance training or exogenous ketones that have anticatabolic effects [193], but this area of investigation remains understudied and controversial.

There is evidence that ketone bodies may protect against muscle loss in catabolic conditions such as sarcopenia, cachexia, and excessive inflammation, potentially through mTORC1 signaling [194]. While still a hypothesis, these previous findings suggest it may be possible to augment preservation of lean tissue during major weight loss through oral ingestion of ketones. In support of this possibility we recently observed that ingestion of a modest does of BHB-salts (12 g day; twice-daily) during a 6-wk ketogenic diet controlled-feeding intervention resulted in a modest reduction in urinary nitrogen excretion compared to placebo (-8%), although this did not translate into a detectable difference in lean body mass determined by DXA [82].

Accumulation of fat in VAT and the liver (NAFLD) are particularly problematic and strongly associate with higher risk for diabetes and cardiovascular disease. Only a few studies have assessed how low-carbohydrate dietary patterns affect VAT using precise imaging technologies. In one study, a 12-wk *ad libitum* ketogenic diet in military-affiliated healthy adults resulted in significant weight loss (7.7 kg) and a 44% reduction in VAT assessed by MRI [195]. In another 12-wk study, patients with NAFLD assigned to a calorie restricted low-carbohydrate diet lost weight (-8.0 kg) and had a 18% reduction in VAT assessed by MRI [196]. Thus, a ketogenic diet may uniquely target VAT [197].

NAFLD is a progressive liver disorder characterized by the accumulation of more than 5% fat in the liver. If left untreated, it can progress to non-alcoholic steatohepatitis, fibrosis, cirrhosis, and liver failure. Hypocaloric low-fat and low-carbohydrate dietary patterns have been demonstrated to reduce liver fat to a similar extent in relatively healthy overweight individuals [198], with more profound effects seen in patients with NAFLD [199]. Some evidence points to superior effects of ketogenic diets [197, 200, 201], likely attributed to both decreased de novo lipogenesis and increased metabolism of fatty acids into the ketogenic pathway [202, 203], both decreasing the availability of fatty acids to accumulate as triglycerides in the liver.

### Effects on Other Pathologic Conditions

The spiraling world-wide problem of obesity, prediabetes/diabetes, and poor metabolic health is exacerbating incidence and complicating treatment of other chronic diseases; and a root cause is poor nutrition. Low-carbohydrate and ketogenic diet interventions are among the most studied of any dietary patterns with regard to metabolic disease with a surge of new and ongoing studies on the horizon. We highlight just a few of the salient conditions closely associated with obesity that are most responsive to low-carbohydrate diets including type 2 diabetes and its complications. We refer the reader to reviews that examine other pathologic conditions that may benefit from low-carbohydrate dietary patterns, especially ketogenic interventions, as a standalone or an adjunct to conventional therapies. These conditions include but are not limited to type 1 diabetes [204], cancer [205], epilepsy [206], Alzheimer’s [207], Parkinson’s [208], multiple sclerosis [209], traumatic brain injury [210], rheumatologic arthritis [211], spinal cord injury [212], and psychiatric disorder [213].

#### Type 2 Diabetes

One of the more salient examples of a therapeutic dietary pattern is that of low-carbohydrate ketogenic diets in the management of T2D. Ketogenic diets were used over a hundred years ago in the treatment of diabetes before the discovery of insulin [214], but only in the last 5 years has this knowledge been officially recognized by professional organizations [215]. Starting with short-term in-patient case studies of people with obese T2D demonstrating striking improvements published in the 1970s [216] and again in the early part of the century [182] to a plethora of longer term outpatient ketogenic diet interventions over the course of the last 20 years [217–219], it is hard not to consider carbohydrate restriction as the default treatment of choice [220]. It is worth noting that low-carbohydrate dietary patterns often reverse T2D while promoting significant weight loss, discontinuing insulin and other glucose-lowering medications, improving cardio-metabolic risk markers, and reducing overall healthcare costs [221].

In one of the longer studies published to date, after 2-yr a group of 262 individuals with T2D were counseled to consume a ketogenic diet using a remote virtual care platform that included medical supervision and medication deprescription, demonstrated T2D reversal in over half the people, defined as having an HbA1c below 6.5% while taking no diabetes medication or only metformin. These individuals also achieved substantial weight loss, improved cardiovascular risk factors, and reduced or eliminated the use of insulin medication [7], with patient retention remaining high (83% at 1; 74% at 2 years).

The chronic complications of T2D that decrease health span, notably heart disease and chronic kidney disease (CKD), should also be alleviated by the improved glucose regulation achieved by a low-carbohydrate dietary pattern, but direct evidence of such a connection is still limited as such studies would likely take many years, if not decades to complete. There is however growing interest in examining the role of low-carbohydrate ketogenic dietary patterns in individuals with heart failure and CKD as discussed next.

#### Heart Failure

Diabetes increases the risk of developing the clinical syndrome heart failure, which affects > 6 million Americans, and is characterized by dyspnea and exercise intolerance. There is very little evidenced-based therapy to guide management of patients with heart failure who maintain preserved ejection fraction, which represents about half of all heart failure patients. Based on several new lines of research, ketogenic-based strategies may be an effective therapy by optimizing myocardial metabolism and cardiac function.

The heart, regardless of its health status, utilizes ketones in proportion to arterial concentration, and the failing heart increasingly relies on ketones [222, 223]. Euketonemia may be a key feature of the ketogenic diet that confers therapeutic effects on the heart [224, 225]. In addition to being a uniquely efficient fuel source for the heart [226], ketones likely have specific cardio-protective and therapeutic effects on the heart [227]. This is noteworthy because the pathophysiology of heart failure is characterized by impaired fatty acid oxidation [228]. In both patients with heart failure and healthy controls, infusion of BHB-salts into the range of nutritional ketosis significantly improved hemodynamic and functional measures of heart function in a dose-response manner [229]. Using exogenous ketone esters, two recent studies also showed that a single serving that achieved euketonemia resulted in improved cardiac function in healthy adults [230, 231].

Another line of evidence connecting ketones to improved cardiovascular disease is the observation that sodium-glucose co-transporter 2 (SGLT2) inhibitor drugs reduce hospitalizations and mortality in patients with diabetes [232, 233], and heart failure patients without diabetes [234]. This class of drugs induces euketoemia increasing production of ketones into the lower range of nutritional ketosis, which has been hypothesized to account for beneficial CVD effects in humans [232]. Cardiovascular benefits of SGLT2 inhibitors may also be attributed to increased diuresis and natriuresis, decreased blood pressure and adiposity, lower glucose and insulin, decreased triglycerides, increased HDL-C, decreased inflammation, and oxidative stress. It is noteworthy that these SGLT2 inhibitor-induced physiological effects mirror the responses to a ketogenic diet.

#### Chronic Kidney Disease

Approximately 40% of adults with T2D will develop CKD and 14% of U.S. adults (~35.5 million individuals) are estimated to have CKD. As many as 9 in 10 adults with CKD remain unaware of their condition, highlighting a significant lack of awareness and diagnosis. CKD is the leading cause of end-stage renal disease (ESRD). Metabolic, hemodynamic, growth, and proinflammatory factors contribute to the pathogenesis of diabetic nephropathy, damaging glomeruli, tubuli, interstitium, and vasculature. Hyperglycemia leads to tissue alteration and fibrosis through advanced glycated end products and glucose metabolism byproducts. Intraglomerular hypertension results from altered arteriolar tone, with insulin playing a role in hyperfiltration. Reducing hyperglycemia is associated with glycemic control benefits and is one method to limit factors in nephropathy development [235].

Considering the therapeutic effects of a ketogenic diet in T2D, it is not surprising that the published research to date also supports a beneficial effect in kidney disease [236]. Polycystic kidney disease (PKD) is a form of CKD with limited therapeutic options that shows remarkable improvement in response to several interventions that achieve euketonemia [237]. In the recently published KETO-ADPKD study, a randomized controlled trial in patients with PKD, a ketogenic diet was shown to decrease body fat and liver fat and increase kidney function compared to a control group [238], and patients rated the ketogenic diet as "highly feasible".

#### Women’s Reproductive Health

Another condition closely associated with obesity and insulin resistance is Polycystic Ovary Syndrome (PCOS), which affects over 1 in 10 women worldwide. Women diagnosed with PCOS are at a greater risk of high blood pressure (30%) [239], insulin resistance (70%) [240], non-alcoholic fatty liver disease (43%) [241], depression (64%) and anxiety (57%) [242], overweight or obesity (38-88%) [240], and even endometrial cancer (270%). These comorbidities contribute to over $5 billion dollars to the annual healthcare burden in the US. Weight loss is strongly advocated for symptom management. However, more extended adherence to carbohydrate-restriction in PCOS appears to offer additional significant benefits. A 12-wk pilot trial demonstrated that a carbohydrate-restriction, without pharmaceutical assistance, positively regulated menstrual cycles to a comparable extent as a mixed diet with pharmaceutically-treated PCOS. Notably, the ketogenic diet exhibited favorable changes in glucose control and body weight [243]. Another single-arm trial spanning 12 weeks, involving 14 women with PCOS, highlighted the feasibility and non-pharmacological efficacy of carbohydrate-restriction interventions. This approach led to improvements in body weight, body composition, insulin sensitivity, cardiometabolic risk, and specific female circulating hormones [244]. In a 24-week study with 11 women enrolled in a carbohydrate-restriction intervention, positive reductions in testosterone, luteinizing hormone: follicle-stimulating hormone, and fasting insulin were observed by the study's end, indicating potential long-term benefits for women with PCOS [245]. A study focusing on nutrient-dense, whole-food carbohydrate-restriction versus fat-restriction reported improvements in weight, BMI, body composition, and blood parameters among pre-menopausal women after six weeks. Interestingly, changes in self-reported menstrual patterns were predominantly noted among carbohydrate-restricted participants, suggesting potential unique effects of nutritional ketosis, independent of weight loss, on female reproductive physiology.

## Benefits of Combining Exercise with Diet

As overviewed in the previous section, nutritional considerations are paramount in the context of major weight loss, but it is important to also consider the role of exercise. It seems reasonable to speculate that regular exercise may promote weight loss, but an equally plausible hypothesis is that a healthy weight enables people to be more active. There may be truth to both perspectives. Surprisingly, when a sedentary person performs an exercise training program the vast majority of studies indicate that regular physical activity, in the absence of dietary intervention, is not effective at promoting clinically significant weight loss in the majority of people [20, 22, 23, 246].

Why the caloric expenditure associated with performing structured exercise fails to translate into a predictable weight loss (i.e., assumes a 3,500 kcal deficit results in a 1-pound fat loss) remains unclear. There is likely a genetic component to how people with excess adiposity translate exercise into weight loss. For example, in a remarkable identical twin metabolic ward study, obese siblings lost a similar amount of weight but only ~20–30% of twin pairs lost the predicted amount of weight in response to a precisely monitored exercise dose (640 kcal/day), whereas some twin pairs lost almost no weight [247]. This is consistent with evidence supporting a restraint on human metabolic rate in individuals who are extremely active [248] and larger studies that show only about a quarter of participants exercising at a high dose do not compensate (i.e., they lose weight in response to exercise) [25]. Ironically, the “compensatory” responses to exercise that contribute to minimal weight loss appear to be more robust in people with greater adiposity [30], making weight loss even more challenging. The mechanisms underlying compensation likely involve an increase in appetite and subsequent caloric intake [26, 27] as well as a decrease in resting energy expenditure [28, 29].

Regardless of the poor effects of exercise alone on weight loss, incorporating regular physical activity into a weight loss plan may have other benefits such as assisting in weight maintenance and improving body composition and health outcomes. We briefly discuss the literature on both endurance/aerobic and resistance/strength training and focus on studies that compared background diets that emphasized low-fat/high-carbohydrate versus low-carbohydrate/high-fat.

### Endurance Exercise

While endurance training alone is a weak weight loss tool for many, it has been shown to slightly augment fat loss and retention of lean mass [249] and is associated with a myriad of health benefits. The majority of studies that examined combined diet interventions and exercise training on weight loss followed nutritional principles consistent with a low-fat/low saturated fat national guidelines-recommended eating plan.

Large multi-site studies of intensive lifestyle diet and exercise studies have shown slightly better outcomes than exercise alone [20]. For example, the Look AHEAD study was an RCT in > 5,000 overweight/obese participants with type 2 diabetes who were randomized to an intensive lifestyle intervention (ILI) consisting of low-fat caloric restriction, physical activity (> 175 min/wk, 10,000 steps/day), and behavior modification or a control group who received standard diabetes education. After 1-yr the ILI group lost significantly more weight than the control group (8.6% vs 0.7%, respectively), but the ILI gained about half that weight back after 4-years. At 8-yr, weight loss was 4.7% for ILI and 2.1% in Controls [44]. The Look AHEAD trial was stopped early because there were no cardiovascular benefits observed in ILI.

Despite a high-intensity of interaction with participants, especially during the weight loss phase, the general failure of Look AHEAD to achieve robust weight maintenance and health benefits may have been attributed to prescribing a diet that heavily emphasized low-fat, especially saturated fat. The participants in Look AHEAD were insulin resistant, which as mentioned in the previous section, manifests as a form of carbohydrate intolerance and is much more responsive to a low-carbohydrate eating pattern. Although speculative, it remains unknown if better results would have been possible if the diet prescription focused on carbohydrate restriction. Preliminary 5-year data demonstrated clinically significant weight loss, cardiometabolic health and blood glucose control improvement, and medication deprescription was shown in a similar population provided intensive education on a low-carbohydrate ketogenic diet [57].

While low-carbohydrate diets have a strong track record in promoting equal or superior weight and fat loss than low-fat diets, there are fewer studies addressing combined low-carbohydrate intake with endurance training. It should be noted that sports nutrition guidelines strongly encourage high-carbohydrate feeding for athletes [21] based on the belief, albeit one that has been repeatedly refuted [250], that frequent carbohydrate feeding is requisite to perform and optimally adapt to training. Several studies have demonstrated at least equal performance with a low-carbohydrate compared to a high-carbohydrate diet if people are allowed to adapt for at least 4-weeks [84, 251]. It has also been observed that the combination of a low-carbohydrate diet and endurance training markedly enhances fat oxidation and often results in weight and fat loss even when prescribed ad libitum [251–253].

### Resistance Exercise

Similar to endurance exercise, resistance training alone is not advocated as a primary method of weight loss [22], but it does augment retention of lean mass during major weight loss, and thereby improves body composition [32] while also promoting numerous health benefits. Again, the majority of diet and resistance training studies examining weight loss were performed in the context of moderate-to-high carbohydrate and low-fat intakes, but a few low-carbohydrate diet interventions have been published recently.

According to recent meta-analyses examining resistance training combined with either a ketogenic or non-ketogenic diet, there was an overall greater loss of body mass, fat mass, percent body fat, and lean body mass in individuals assigned to a ketogenic diet [254, 255]. While the decrease in body fat is desirable, the usual interpretation of decreased lean mass in response to a low-carbohydrate, especially ketogenic, diet is negative [254], attributed to a loss of contractile proteins secondary to reduced insulin secretion and anabolic signaling. However, is the loss of lean tissue on a ketogenic diet during training maladaptive or even a real phenomenon? Four unappreciated points are warranted to properly contextualize the lean mass response to a ketogenic diet.

First, loss of lean mass in response to a ketogenic diet is often accompanied by an overall greater loss in body mass, such that body composition (i.e., percent body fat) improves [254, 255]. Second, many ketogenic diet studies fail to provide adequate mineral requirements, especially the increased sodium requirements and adequate potassium unique to the ketogenic diet, which in turn compromises muscle protein anabolism [192]. Third, loss of a certain amount of lean tissue is expected whenever there is significant fat loss because adipose tissue consists of ~5% protein and 15% water by weight. Fourth, decreased lean mass is at least partially an artifact of greater water loss on a ketogenic diet [83] vis-a-vis the renal natriuretic and diuretic effect of carbohydrate restriction, as well as water loss associated with breakdown of intramuscular glycogen. As previously mentioned, DXA detects water as lean mass, and therefore is subject to specious findings in the context of even modest fluid shifts [256, 257].

A relevant example comes from a resistance training study that demonstrated participants consuming a 10-wk ketogenic diet had numerically lower (but not statistically different) DXA-determined lean body mass as a mixed diet group (2.2% vs 4.4%, respectively) [258]. An interesting feature of this trial was that the ketogenic diet group reintroduced carbohydrate from Week 10 to 11. During this 1-wk period they demonstrated a tripling of lean body mass such that the delta increase from baseline to Week-11 was greater in the ketogenic diet group (7.3% vs 3.6%). One week of carbohydrate feeding would be expected to increase insulin, potentiating an anti-diuretic effect, as well as promoting storage of glycogen and water in muscle cells; all of which would be detected as lean mass by DXA [256, 257]. This effect, operating in reverse, partially explains the loss of lean mass in many ketogenic diet and resistance training studies. This interpretation seems even more probable considering that functional measures of strength and power in response to resistance training are not compromised by a ketogenic diet [195, 258, 259].

In fact, the combination of a carbohydrate-restricted diet and resistance training optimizes body composition resulting in the greatest decreases in whole body adiposity and percent body fat [195, 260], including significant loss of visceral fat, while shifting skeletal muscle mitochondrial function and efficiency towards fat oxidation in conjunction with improved insulin sensitivity [261].

In summary, endurance and resistance training are not effective primary methods of weight loss for most people, but research supports a consistent benefit of exercise when combined with diet, especially resistance training, on weight maintenance, lean mass and physical performance. Although there is more debate surrounding the optimal dietary macronutrient distribution, there is preliminary evidence supporting the combination of a low-carbohydrate diet with resistance training to maximize fat loss and retention of lean while improving many cardiometabolic risk factors.

## Limitations

The high prevalence of obesity and associated disorders is a major problem many humans are facing. No single weight loss approach is suitable for all people with excess adiposity given the heterogeneity of response to diet and exercise. A major limitation in the literature is the lack of attention on identifying methods of tailoring approaches that are most effective for the individual. There are also inherent challenges in conducting diet interventions and exercise training studies owing to the difficulties in accurately monitoring and achieving long-term compliance and adherence. There is also the high cost and complexity of conducting long-term studies that has limited the number of diet options that can be studied to mostly low-fat eating patterns. No federally funded studies have examined the effects of a low-carbohydrate eating pattern for more than a couple years. Other limitations relate to the need for increased attention on assessing body composition in weight loss studies, which is generally not the case for many surgical and drug studies. There is a challenge of accurately quantifying lean and fat mass during weight loss given the potential for large artifactual errors associated with some body composition assessment methods including DXA.

## Summary

In summary, an impressive amount of scientific evidence now supports low-carbohydrate dietary patterns that contain moderate protein (1.2 to 2.0 g/kg reference weight) from food sources with high biological value as effective for promoting clinically meaningful decreases in adiposity without unwanted muscle mass loss. Low carbohydrate dietary patterns, especially those that achieve a natural state of *euketonemia*, also improve many pathologic conditions, notably type 2 diabetes and other conditions related to insulin resistance. The landscape of low-carbohydrate and ketogenic diet research now extends beyond the well-established research in epilepsy, weight loss, and type 2 diabetes to new frontiers that include conditions such as type 1 diabetes, cancer, epilepsy, Alzheimer's, Parkinson's, multiple sclerosis, traumatic brain injury, rheumatologic arthritis, spinal cord injury, and psychiatric disorders. Whether these conditions demonstrate the same robust therapeutic effects remains to be seen, but we believe these investigations hold the potential to uncover additional beneficial metabolic and physiological effects that broaden our understanding of therapeutic nutrition. As the scientific community delves into these emerging areas, collaboration between researchers, healthcare practitioners, and policymakers is crucial to translate findings into accessible and effective interventions that promote individual and global well-being.

## Data Availability

No datasets were generated or analysed during the current study.
